# 4-((1E)-2-(5-(4-hydroxy-3-methoxystyryl-)-1-phenyl-1H-pyrazoyl-3-yl) vinyl)-2-methoxy-phenol) (CNB-001) Does Not Regulate Human Recombinant Protein-Tyrosine Phosphatase1B (PTP1B) Activity *in vitro*

**DOI:** 10.4172/2155-9562.1000232

**Published:** 2014-09-29

**Authors:** Paul A Lapchak, Jacqueline A Lara, Paul D Boitano

**Affiliations:** 1 Department of Neurology, Cedars-Sinai Medical Center, Advanced Health Sciences Pavilion, Los Angeles, USA; 2 Department of Neurosurgery, Cedars-Sinai Medical Center, Advanced Health Sciences Pavilion, Los Angeles, USA

**Keywords:** Curcumin analog, Neuroprotection, Metabolic disorder, Neurotrophic, Phosphatases, Pleiotropic

## Abstract

Protein-Tyrosine Phosphatase1B (PTP1B) is a negative regulator of the insulin signaling pathway and is a potential therapeutic target for treatment of type 2 diabetes, cardiovascular disease, metabolic syndrome and cancer. It has been postulated that CNB-001 [4-((1E)-2-(5-(4-hydroxy-3-methoxystyryl-)-1-phenyl-1H-pyrazoyl-3-yl) vinyl)-2-methoxy-phenol)] may regulate PTP1B activity suggested by a computer-based active site docking recognition model. This possibility was studied using a human recombinant PTP1B assay, and a phospho-peptide fragment of the insulin receptor β subunit domain (IR5). The positive control, suramin, inhibited PTP1B with an IC50 (half minimal (50%) inhibitory concentration) value of 16.34 µM; CNB-001 did not affect enzyme activity across the range of 1nM–0.1mM. This study suggests that PTP1B inhibition is not involved in the beneficial effects of CNB-001 in obese type 2 diabetic mice.

## Introduction

CNB-001 [4-((1E)-2-(5-(4-hydroxy-3-methoxystyryl-)-1-phenyl-1H-pyrazoyl-3-yl)vinyl)-2-methoxy-phenol)], is an important first in class pleiotropic drug candidate being developed to treat neurodegenerative diseases such as acute ischemic stroke and Alzheimer’s disease [1–4]. Using phenotypic screening assays directed against some of the exacerbating mechanisms (mitochondrial dysfunction, oxidative stress glutamate toxicity) underlying deficits resulting from activation of the stroke cascade, CNB-001 has been shown to support cell survival [5], CNB-001 also a potent 5-lipoxygenase inhibitor (5-LOX) [4], anti-apoptotic and antioxidant [6], a negative regulator of inflammation [down-regulates, 5-LOX, cyclooxygenase-2 (COX-2), interleukin-6 (IL-6)] [3,7,8], and an activator of brain-derived neurotrophic factor (BDNF) and its signaling pathways [3]. The pleiotropic nature of the drug makes an optimal candidate for cellular protection and repair for a variety of diseases that currently have limited treatment options [2,3,7,9–16].

We recently studied the pharmacological effects of CNB-001 in a diabetic mouse model (7), a model of obesity-associated insulin resistance induced by a high fat diet. The study found that CNB-001 effectively attenuated or reversed many of the deleterious changes including weight gain, serum triglycerides and serum IL-6 levels. Moreover, in obese mice, CNB-001 increased energy expenditure and high-fat insulin resistance, glucose tolerance and reversed gastrocnemius muscle deficits (i.e.: 2-deoxyglucose uptake). Importantly, in the study, Panzhinskiy et al. [12] hypothesized that CNB-001 may regulate Protein-Tyrosine Phosphatase1B (PTP1B) based upon the best fit of the molecule using computer-based active site docking recognition. PTP1B is a negative regulator of the insulin signaling pathway and is a potential therapeutic target, in particular for treatment of type 2 diabetes [12–16]. Since preliminary studies suggested that CNB-001 may potentially bind to PTP1B and regulate PTP1B activity, an effect of CNB-001 on the phosphatase may explain some of the beneficial effects that were observed.

Thus, using a human recombinant PTP1B assay, and a phospho-peptide fragment of the insulin receptor β subunit domain, we tested the hypothesis that CNB-001 interacts with the enzyme. In this controlled study, we used suramin as the positive control inhibitor [17].

## Materials and Methods

### Drug preparation

CNB-001 (AQ BioPharma Co. Ltd, Shanghai, China) was dissolved in reagent grade dimethyl sulfoxide (DMSO) (Sigma-Aldrich, Saint Louis, MO) for assay.

### Enzyme assay

The PTP1B Colorimetric Assay Kit was purchased from Enzo Life sciences International Inc., Plymouth Meeting, PA. The assay kit contained recombinant PTP1B enzyme (residues 1–322 M.W. 37,400) expressed in E. coli. The phosphopeptide substrate (IR5) used in the sensitive assays contains the amino acids 1142–1153pY1146 (MW-1703 kDa), a sequence from the insulin receptor β subunit domain that must be autophosphorylated to achieve full receptor kinase activation. This "activation loop" is the target of several protein phosphatase regulators of insulin signaling, including, notably, PTP1B. The enzyme has an estimated Km of 85 µM for the PTP1B phospo-peptide substrate (IR5) which is used to measure PTP1B activity.

### Methods

Since the assay endpoint is the measurement of free phosphate, a phosphate standard curve was prepared per the manufacturers protocol; six concentrations of inorganic phosphate were established in duplicate wells: 0, 0.25 nmol, 0.5 nmol, 1.0 nmol, 2.0 nmol and 3.0 nmol by adding the appropriate amounts of assay buffer and phosphate standard. All test sample/inhibitor assays were performed following the protocol, with variances only in the concentrations of the reagents (as described): PTP1B enzyme was prepared for all assays to allow for a final amount of 2.5 ng/well and IR5 was prepared to have a final assay concentration of 75 µM. Briefly; 35 µl of 1X assay buffer (100 mM MES, pH 6.0, 300 mM NaCl, 2 mM EDTA, 2 mM DTT, 0.1% NP-40) was added to each well and incubated for 15 minutes at 39°C; 10 µl of CNB-001 or DMSO as baseline control (final concentrations 1.0 mM, 0.1 mM, 10 µM, 1µM, 10nM and 1.0nM) were added to each well followed by 5 µl of PTP1B enzyme (2.5ng per well) dilution. In experiments using suramin, it was dissolved in water, and 10µl of suramin or water were added to each well. Reactions were initiated by adding 50µl of IR5 substrate (final concentration 75µM) and the plate was incubated for 30 minutes at 39°C. Reactions were then terminated by adding 25µl of the provided phosphate detection reagent (Red Reagent) and wells were agitated gently to mix. Color was allowed to develop for 25–30 minutes, and absorbance was read at 620 nm using a Spectra Max M2 spectrophotometer (Molecular Devices, Sunnyvale, CA).

### Results

In this controlled study, we used suramin, a reversible and competitive inhibitor of PTP1B to establish the characteristics of the assay [17]. Suramin has a Ki of approximately 5.5 µM and an IC50 value in the range of 9.5–11 µM for PTP1B inhibition, as reported in the literature (17). In the suramin study, the standard curve was linear with a regression equation of y = −0.013 + 0.2086 B and a regression analysis fit of R2= 0.9972. Thus, phosphate product amounts could easily be calculated.

**Figure 1:**
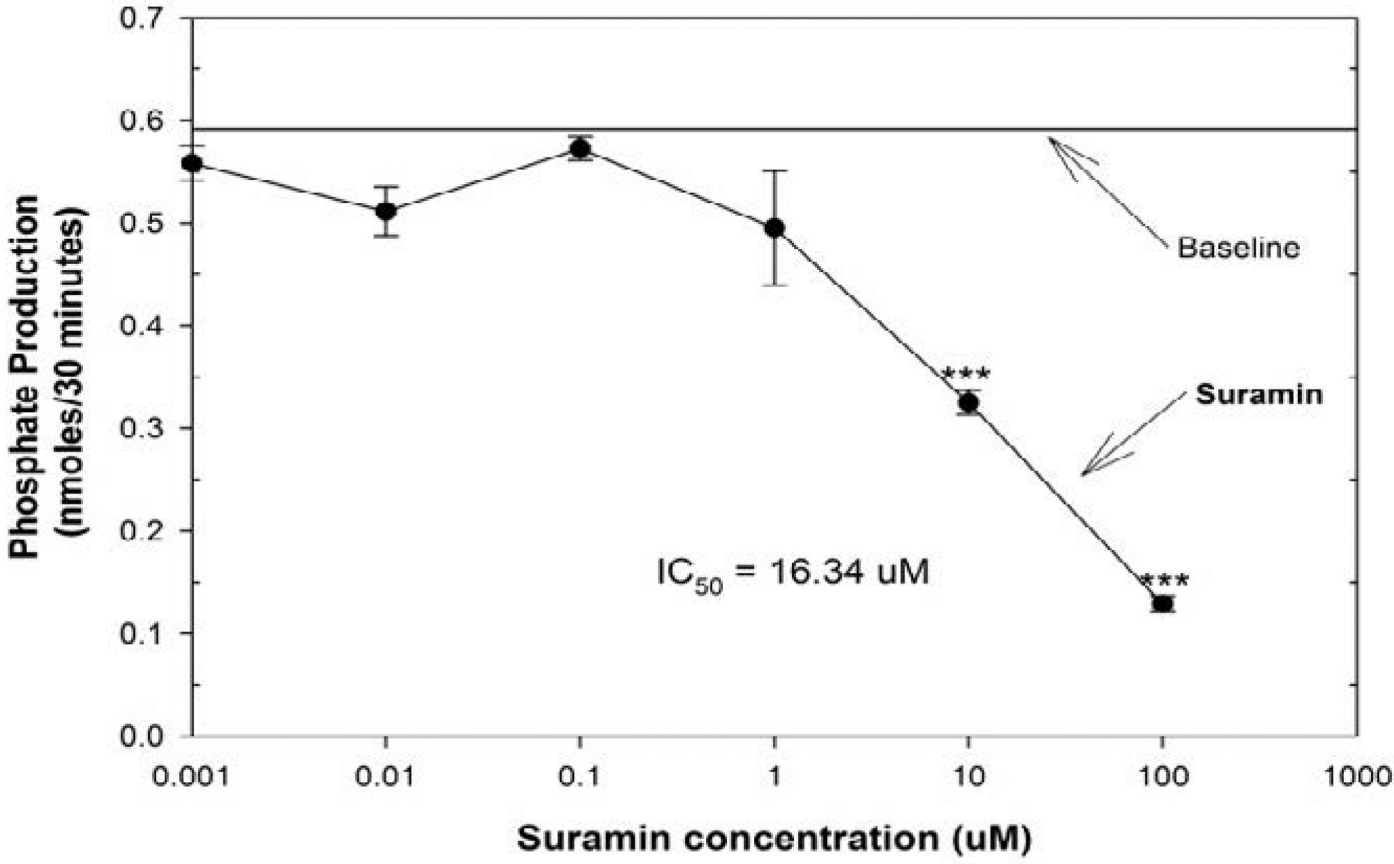
Suramin inhibits PTP1B with an IC50 of 16.34 µM; PTP1B assays were run in triplicate with either buffer or suramin (0.1mM–1nM). Significantly different (***p<0.01) from baseline enzyme activity levels measured by phosphate product produced over 30 minutes. IC50 value is calculated inhibitory concentration required for 50% enzyme activity inhibition compared to baseline control. Paired-t-test was used for statistical analysis (Graph Pad Inc.).

In Figure 1, we provide data demonstrating the effect of suramin on PTP1B activity. Using suramin concentration of 1.0-nM–0.10 mM we constructed an inhibition curve, we show that low doses of suramin (1.0 nM to 0.1 µM) had no effect on PTP1B activity. However, higher doses of suramin inhibited PTP1B and phosphate production. In our assay, the IC50 (half minimal (50%) inhibitory concentration) value was 16.34 µM for 50% PTP1B inhibition.

**Figure 2:**
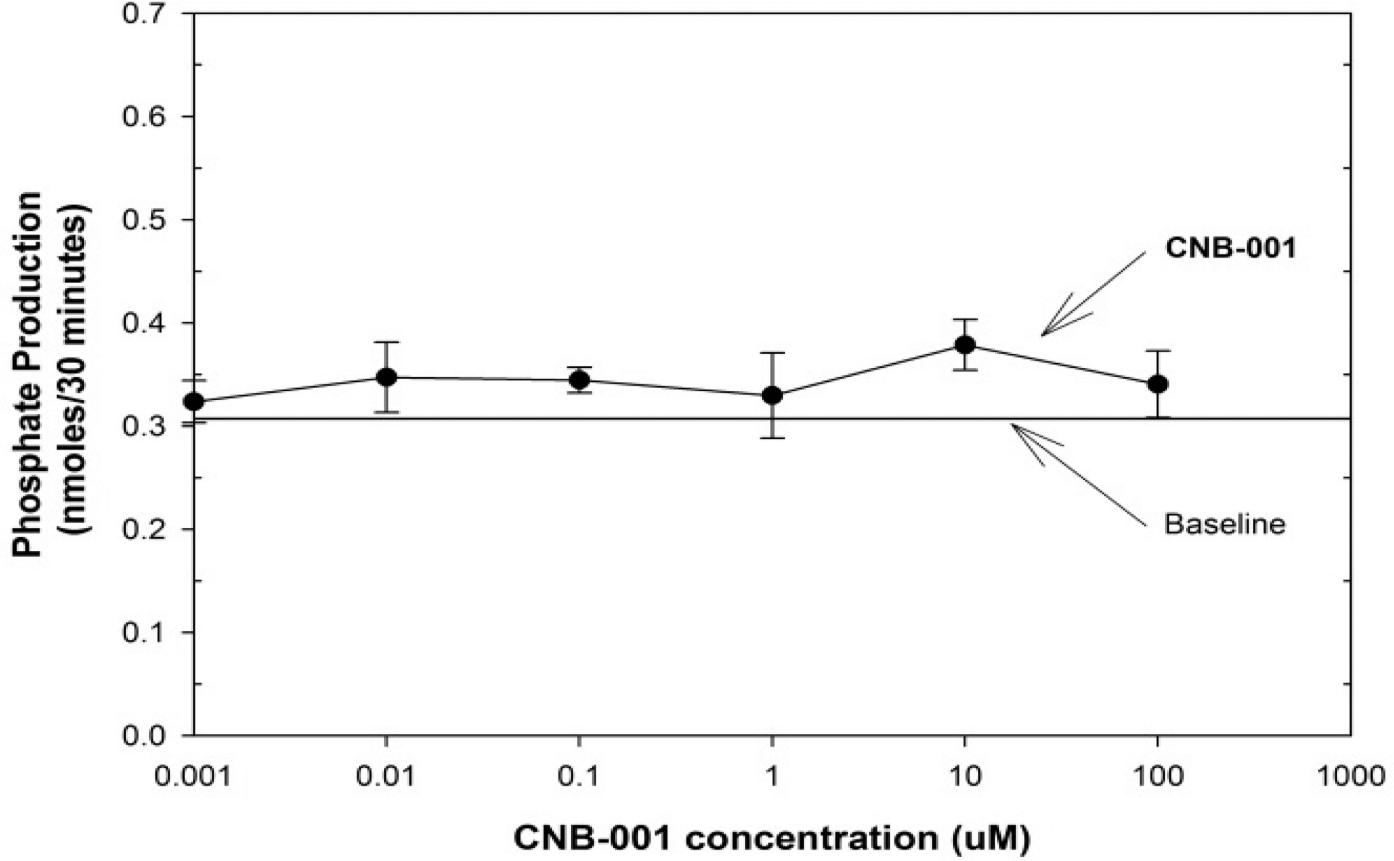
CNB-001 does not affect PTP1B activity in vitro; PTP1B assays were run in triplicate with either DMSO of CNB-001 (0.1mM–1nM). Activity measured in the presence of CNB-001 was not significantly different (p>0.05) from baseline enzyme activity levels measured by phosphate product produced over 30 minutes. Baseline was lowered by the presence of DMSO in the assay. Paired-t-test was used for statistical analysis (Graph Pad Inc.).

In Figure 2, we provide data for the effects of CNB-001 on PTP1B across the concentration range of 1.0-nM–0.10 mM. In the CNB-001 study, the standard curve was linear with a regression equation of y = −0.010 + 0.1349 B a regression analysis fit of R2= 0.9821. Thus, even in the presence of DMSO, phosphate product amounts could easily be calculated. Note that in the presence of DMSO, the amount of phosphate detected using the RED reagent was reduced compared to the phosphate detected in the absence of DMSO (i.e.: buffer alone). Nevertheless, for each drug, we constructed standard curves using the vehicles used to solubilize each drug so that regression analysis could be conducted. In this assay, CNB-001 did not have any effect on PTP1B activity.

## Conclusion

We tested the hypothesis that CNB-001 interacts with PTP1B to produce the beneficial effects observed in obese diabetic mice. Using a specific recombinant human PTP1B assay, under conditions recommended by the manufacturer, we found that CNB-001 did not regulate PTP1B activity, but suramin effectively inhibited enzyme activity with an IC50 similar to that previously documented in the literature [17]. Based upon this data, it does not appear that CNB-001 regulates PTP1B activity, and CNB-001 is not an inhibitor of PTP1B.
